# Two conserved oligosaccharyltransferase catalytic subunits required for N-glycosylation exist in *Spartina alterniflora*

**DOI:** 10.1186/s40529-015-0111-9

**Published:** 2015-11-11

**Authors:** Luyi Jiang, Xin Zhu, Jinmei Chen, Deyue Yang, Changfang Zhou, Zhi Hong

**Affiliations:** School of Life Sciences, Nanjing University, 163 Xianlin Ave., Nanjing, 210023 Jiangsu China

**Keywords:** N-glycosylation, STT3A, Salt tolerance, *Spartina alterniflora*

## Abstract

**Background:**

Asparagine (N)-linked glycosylation is one of the most crucial post-translational modifications, which is catalyzed in the lumen of the endoplasmic reticulum (ER) by the oligosaccharyltransferase (OST) in eukaryotic cells. Biochemical and genetic assay leads to the identification of the nine subunits (Ost 1–6, Stt3, Swp1 and Wbp1) of the yeast OST and in which Stt3p is proposed playing a central and conserved role in N-glycosylation. Two STT3 isoform genes, *STT3A* and *STT3B*, exist in the plant and mammal genomes. OST with different catalytic STT3 isoforms has different enzymatic properties in mammals. The mutation of *STT3A* in *Arabidopsis thaliana* causes a salt hypersensitive phenotype the inhibited root growth and swollen root tips suggesting protein N-glycosylation is indispensable for plant growth and development. *Spartina alterniflora* is widely used for shoreline protection and tidal marsh restoration due to the strong salt tolerance although the exact molecular mechanism is little known. To explore the possible biological roles of N-glycosylation in plant adaptive resistance to salinity stress, we cloned the *STT3* genes from *S. alterniflora* and heterogenously expressed them in *Arabidopsis* mutant to observe the functional conservation.

**Results:**

*SaSTT3A* and *SaSTT3B* genes were cloned from *Spartina alterniflora*. *SaSTT3A* genomic sequences spanned over 23 exons and 22 introns, while *SaSTT3B* had 6 exons and 5 introns. The gene structures of both genes were conserved among the analyzed plant species. Subcellular localization and transmembrane structure prediction revealed that these two genes had 13 and 11 transmembrane helices respectively. The functional complementation in which the cDNA of *SaSTT3A* and *SaSTT3B* driven by CaMV 35S promoter completely or partially rescued *Arabidopsis* *stt3a*-*2* mutant salt-sensitive phenotype, indicating *STT3A* functions conservatively between glycophyte and halophyte and N-glycosylation might be involved in plant resistance to salinity.

**Conclusions:**

Two STT3 isoform genes, *SaSTT3A* and *SaSTT3B*, were cloned from *S. alterniflora* and they were evolutionally conserved at gene structure and coding sequences compared with their counterparts. Moreover, *SaSTT3* genes could successfully rescue *Arabidopsis stt3a*-*2* salt-sensitive phenotype, suggesting there exists a similar N-glycosylation process in *S. alterniflora.* Here we provided a first piece of evidence that the N-glycosylation might be involved in salt tolerance of halophyte.

**Electronic supplementary material:**

The online version of this article (doi:10.1186/s40529-015-0111-9) contains supplementary material, which is available to authorized users.

## Background

Salinity is a major constraint adversely affecting plant growth, development and productivity. Severe salt stress can even threaten the survival of plants. By now, salinity has become a main abiotic stress limiting crop production worldwide, affecting nearly 40 percent of agricultural lands located in arid and semi-arid areas (Mittler 2006; Roy et al. 2014). Although many signaling components and molecular mechanisms have been identified which largely extend our understanding of plant tolerance against salt stress, the utilization of the knowledge in crop improvement leaves much to be desired since salt stress induces the genetic and physiological changes at all levels of organization in plants and the salinity tolerance, in common with other environmental stresses, relies on the interactions among multiple genes (Arraouadi et al. 2012; Cortina and Culiáñez-Macià 2005; Flowers and Colmer 2015). Simply expressing one or several genes usually has no obvious improvement on the salt tolerance of transgenic crops.

*Spartina alterniflora* Loisel (smooth cordgrass), a gramineous halophyte, can survive in as high as two fold strength of seawater (Niranjan Baisakh and Parami 2006) and is believed owning all possible mechanisms of salt tolerance, existence of salt glands, decrease in osmotic potential, biosynthesis of compatible solutes, ion exclusion and redistribution (Baisakh 2011; Zandkarimi et al. 2015; Touchette et al. 2009). The most important is that *S. alterniflora* shares 80–90 % similarity with rice on the basis of their DNA and protein sequences (Niranjan Baisakh and Varadwaj 2008). Some genes from *S.alterniflora* could confer salt tolerance in transgenic plants, like rice or *Arabidopsis* (Karan and Subudhi 2012; Baisakh et al. 2012; Baisakh 2014). These characters make *S. alterniflora* an excellent model to explore the mechanisms of plant tolerance against salt stress.

Asparagine (Asn/N)-linked glycosylation is an important posttranslational modification and plays a crucial role in protein folding and stability, cell–cell recognition, signaling, and other cellular processes in eukaryote (Nguyen et al. 2011; Rayon et al. 1998; Schwarz and Aebi 2011; Breitling and Aebi 2013). The reaction is catalyzed in the lumen of the endoplasmic reticulum (ER) by the oligosaccharyltransferase (OST) that transfers a preassembled tetradecasaccharide Glc_3_Man_9_GlcNAc_2_ (Glc, Man and GlcNAc for glucose, mannose, and N-acetylglucosamine, respectively) from a dolichol-linked oligosaccharide donor to Asn residue in the N-X-S/T motif on nascent peptides, where X can be any amino acid except proline (Knauer and Lehle 1999; Mohorko et al. 2011; Yan and Lennarz 2005). Biochemical and genetic assays reveal OST is an octamer and in which STT3 (STAUROSPORIN AND TEMPERATURE SENSITIVE 3) (Yoshida et al. 1992) is proposed playing a central and conserved catalytic role in N-glycosylation based on the fact that archaebacterial genomes contain recognized homologs of only the STT3 subunit (Silberstein and Gilmore 1996; Wacker et al. 2002; Lizak et al. 2011). In yeast, STT3p contributes to substrate recognition and part or all of the OST active site formation (Karaoglu et al. 1997; Spirig et al. 1997; Yan and Lennarz 2002). Two isoforms of *STT3*, *STT3A* and *STT3B*, exist in the plant and mammal genomes. OST with different catalytic STT3 isoform has different enzymatic properties in mammals (Kelleher et al. 2003). In plant, Koiwa et al. reported that *AtSTT3A* and *AtSTT3B* are functionally redundant in regulating plant growth and development in *Arabidopsis thaliana* since deletion of both genes resulted in gametophytic lethality (Koiwa et al. 2003). Interestingly, *STT3A* seems more functionally important than that of *STT3B* because the loss of function of *STT3A* causes a salt hypersensitive phenotype but no growth defects are seen in *STT3B* knockout line, indicating *STT3A* subunit isoform may mediate specific protein glycosylation during salinity stress.

In current study, we described the characterization of sequence of *STT3A* and *STT3B* from *S. alterniflora*. Both genes shared high similarity with their counterparts according to the multiple alignments analysis. Furthermore, the functional complementation test was conducted to observe whether *SaSTT3A* and *SaSTT3B* could rescue *A. thaliana stt3a*-*2* salt hypersensitive phenotype. Our results showed SaSTT3A and SaSTT3B are structurally and functionally conserved among halophyte and glycophyte, suggesting a similar N-glycosylation process exists in halophyte. Our work opened a door to understanding of the functional importance of N-glycosylation in plant tolerance against salinity in *S. alterniflora*.

## Methods

### Plants growth and stress treatments

*A. thaliana* ecotype Columbia (Col-0) was used as wild-type control. *stt3a*-*2* (CS800052, ecotype Columbia) mutant was previously described (Koiwa et al. 2003). Seeds were surface sterilized with 75 % (v/v) ethanol containing 0.05 % (v/v) Tween 20 for 15 min, followed with 95 % (v/v) ethanol for 2 min and then were sown on ½ Murashige and Skoog (½ MS) medium. The seeds were treated at 4 °C for 2 days and then incubated at 24 °C for 2 weeks under long-day condition (16 h light/8 h dark). The seeds of *S. alterniflora* were collected at Nature Reserve of Sheyang, China, and stored for 120 days at 4 °C. After cold treatment, the seed were placed on two pieces of filter paper in petri dishes merged in water or artificial seawater for germination. For root length measurement under high salt conditions, seeds were germinated on ½ MS medium for 3 days and then transferred to MS medium (1× MS salts, 30 g/L sucrose, and 16 g/L agar, pH 5.7) supplemented with 160 mM NaCl in vertical plates.

### DNA and RNA isolation

Plant tissues was frozen in liquid nitrogen and grounded into fine powder. Total genomic DNA was extracted using the protocol described by Dellaporta (Stephen et al. 1983). RNA was isolated from plant tissue using TRIzol reagent (Invitrogen, USA) following the manufacturer’s instructions. mRNAs were isolated using the PolyATtract^®^ mRNA Isolation System III (Promega) following the supplier’s protocol. The quality and content of DNA and RNA in samples were examined using agarose gel electrophoresis or spectrophotometer by measuring absorbance at 260 and 280 nm.

### Cloning of *SaSTT3A* and *SaSTT3B* cDNA and genomic sequences

The cDNAs were synthesized and amplified using SMARTScribe Reverse Transcriptase (Clontech, USA) following the method described previously (Matz et al. 1999). In terms of the sequence alignment among the closely related species, specific primers were designed, together with the long arbitrary degenerate (LADs) primers for hi-TAIL PCR and adaptor primers (UPML and UPMS) for 5′ and 3′ RACE to obtain the *SaSTT3A* and *SaSTT3B* cDNA sequences. The position of the primers was depicted in Additional file 1: Figure S1 and the sequences were listed in Additional file 1: Table S1.

cSaSTT3A-1F/1R and cSaSTT3A-2F/2R were used to get 2 parts of the conserved sequences. cSaSTT3A-1F/R2 were used to amplify the midstream of the cDNA of *SaSTT3A*. SaSTT3A-3GSPF1/F2 and ASP were employed as the first and the second round for 3′ RACE. SaSTT3A-SPR0/SPR1/SPR2 and LADs were used to get the 5′ end. SaSTT3A-5GSPR1/R2 together with the adaptor primer (UPM) were used for 5′ RACE of *SaSTT3A*.

cSaSTT3B-1F/1R were used to get the first segment of *SaSTT3B* cDNA and cSaSTT3B-2F/2R for the second part, cSaSTT3B-3F1/3F2/3R for the third part. Then, SaSTT3B-SPR0/SPR1/SPR2 and LADs were used to get the 5′ end unknown sequence. SaSTT3B-5GSPR1/2, SaSTT3B-3GSPF1/2, as the first round and second round of 5′ RACE or 3′ RACE, were used with the adaptor primer (UPM) and ASP to obtain both ends, respectively.

The PCR cycling of 5′ RACE conditions was one cycle of 94 °C for 5 min, 29 cycles of 94 °C for 20 s, 68 °C for 20 s and 72 °C for 50 s, followed by one cycle of 72 °C for 7 min. The 3′ RACE was performed using the following conditions: 94 °C for 5 min, followed by twenty-nine cycles of 94 °C for 20 s, 60 °C for 20 s, 72 °C for 1 min, and then a final extension step at 72 °C for 7 min. The PCR cycling of hi-TAIL PCR was previously described (Liu and Chen 2007). The gel-purified fragments were cloned into pMD18-T vector (TaKaRa, Japan) and transformed into E. coli strain TOP10 competent cells. The positive clones were screened by PCR and then subjected to sequencing.

The strategy for genomic sequences was the same as that of cDNAs and the primer sets were listed in Additional file 1: Table S2.

### Sequence analysis

Protein prediction was performed using software at the ExPASy Bioinformatics Resource Portal (http://www.expasy.org/). The putative transmembrane sequences were identified via the TMpred server. The topology model of the cloned SaSTT3A and SaSTT3B was predicted with TOPO2. The N-glycosylation sites were calculated using NetNGlyc 1.0 Server. Multiple alignments were generated at the web site. Identical residues are shaded in black, and residues with amino acid similarity are shaded in light gray using boxshade server. The phylogenetic tree was constructed based on the deduced amino acid sequences using the software MEGA (version 5.1) with the method of Neighbour-Joining (NJ) algorithm.

### Expression of *SaSTT3A* and *SaSTT3B* and plant transformation

Total RNAs were extracted from *S. alterniflora* leaves using TRIzol reagent. 1 μg RNA was reverse-transcribed with AMV Reverse Transcriptase (TaKaRa, Japan) according to the standard protocols. The construction of pCHF1:SaSTT3A and pCHF1:STT3B was described as follows. The full-length *SaSTT3A* or *SaSTT3B* cDNA was amplified using primers SaSTT3A-KpnI-F, SaSTT3A-SalI-R, or SaSTT3B-EcoRI-F, SaSTT3A-PstI-R and cloned into the binary vector pCHF1 (Fankhauser et al. 1999) that carries the 35S promoter and the pea (*Pisum.sativum*) RbcS-E9 terminator. Alternatively, AtSTT3A promoter sequences were amplified into pBluescript vector and the site-directed mutagenesis was performed to create an Afl II site which permits cDNA sequences of SaSTT3A and SaSTT3B being cloned into the vector between it and NheI sitse. A new KpnI site in SaSTT3B and the mutated nucleotides in the *SaSTT3* genes during the earlier step were recovered using site-mutagenesis, finally the full length *SaSTT3A* and *STT3B* containing the AtSTT3A promoter sequences were subcloned into pPZP222 vector at PstI/EcoRI and KpnI sites, respectively. The sequences of primers were listed in Additional file 1: Table S3. Genetic transformation of *Arabidopsis stt3a*-*2* through *Agrobacterium tumefaciens* harboring the plasmids was conducted by vacuum infiltration as described by Clough and Bent (Clough and Bent 1998). T1 transformants were selected on ½ MS medium containing 90 mg/L gentamycin and 100 mg/L timentin and then screened on the medium containing 150 mM NaCl. For phenotypic observation, the T3 transgenic seedlings expressing *SaSTT3A* or *SaSTT3B* were subjected to 150 mM NaCl stress medium after germination and grew for 10 days. Plants transformed with empty vector as a negative control.

For detection of target gene expression, primer sets of cSaSTT3A-spe-F/R and cSaSTT3B-spe-F/R were used to analyze positive transcripts of *35S:SaSTT3A/stt3a*-*2* and *35S:SaSTT3B/stt3a*-*2* seedlings. RT-PCR products were analyzed via 1.2 % agarose/EtBr gel electrophoresis. Actin gene as an internal loading control. Quantitative RT-PCR was performed with the gene-specific primers listed in Additional file 1: Table S3 using a 7300 Real-Time PCR System (Applied Biosystems, USA). Thermal cycle conditions consisted of an initial denaturation incubation at 95 °C for 5 min followed by 35 cycles of alternating 95 °C incubations for 20 s, 58 °C incubations for 20 s and 72 °C incubations for 30 s. Fluorescence was detected after every 72 °C extension incubation. The levels of mRNA of all the genes were normalized to α-actin mRNA level using the 2^−ΔΔCt^ method.

### Western blot analysis

The frozen Arabidopsis seedlings were grounded into powder and dissolved in 2XSDS sample buffer,mixed well and boiled for 5 min. After centrifugation at 13000 g for 10 min, supernatants were used for immunoblot analysis. Total soluble protein samples were separated on 10 % SDS-PAGE gel and then transferred onto PVDF membrane. PVDF membrane was incubated in blocking solution (5 % milk powder in TBS buffer supplemented with 0.05 % Tween 20) for 40 min and then changed PDI antibodies (Rose Biotechnology) for immune blot. After wash and the incubation with the secondary antibody, the detection was performed using Western Chemiluminescent HRP substrate (ECL) kit (Millipore). The chemiluminescent signals were recorded on x-ray films to get the signals.

## Results

### Cloning of cDNA sequences of *SaSTT3A* and *SaSTT3B*

The full length of the CDS sequences of *SaSTT3A* and *SaSTT3B* were amplified from total cDNA pool using the primers located in the consensus region of STT3 homologs and the cloning procedure of the cDNAs were described in *Materials and Methods* and the primers used for cloning were listed in Additional file 1: Table S1. A length of 2361 bp cDNA sequences was cloned for *SaSTT3A* and was predicted encoding 786 amino acids. *SaSTT3B* CDS had 2163 bp and encoded 720 amino acids. The sequences of untranslated region (UTR) were also studied. The length of 5′ UTR in *SaSTT3A* and *SaSTT3B* was 62 and 99 bp, respectively. Interestingly, two types of 3′ UTR transcripts were found in both genes, that was 266 or 273 bp in *SaSTT3A* and 227 or 343 bp in *SaSTT3B.* The alternative splicing of 3′ UTR suggested the expression of these two genes were probably regulated at a post-transcriptional level.

**Fig. 1:**
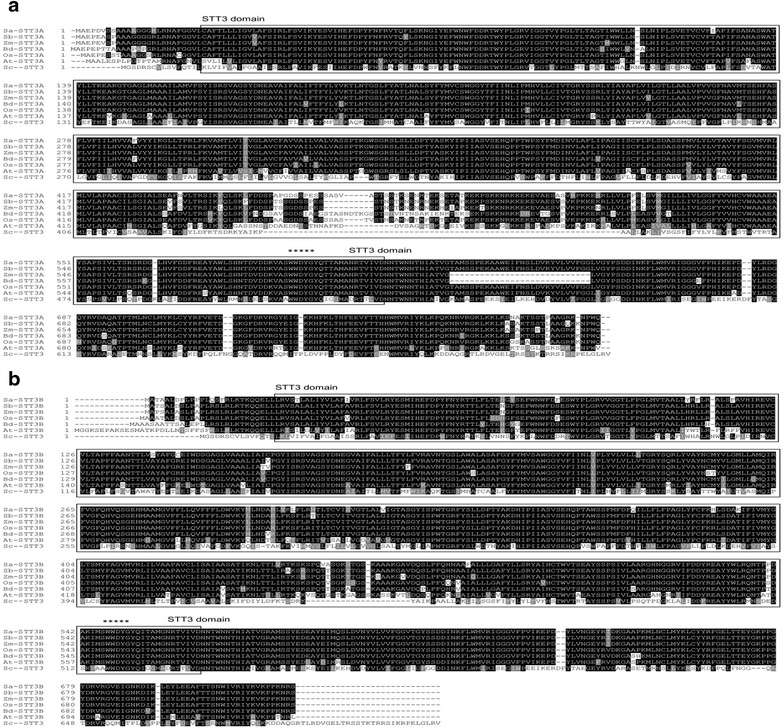
The alignment of the deduced amino acid sequences of SaSTT3A and SaSTT3B with their homologs. The multiple alignments among SaSTT3A (**a**) or SaSTT3B (**b**) with their homologs were built by ClustalW2 server (http://www.ebi.ac.uk/Tools/msa/clustalw2/). Identical residues ≥4 sequences are shaded *black*, while similar residues were shaded in *gray* using the *Boxshade* server at http://www.ch.embnet.org/software/BOX_form.html. The conserved STT3 domain was *boxed* and the characteristic motif of WWDYG was marked with *asterisks* at the *top* of the alignment block. The accession number of the selected STT3 proteins are indicated as follows: Sb, *Sorghum bicolor*, STT3A: XP_002441399.1, STT3B: XP_002448765.1; Zm, *Zea mays*, STT3A: AFW78839.1, STT3B: NP_001131374.1; Os, *Oryza sativa*, STT3A: NP_001056068.1, STT3B: NP_001054248.1; Bd, *Brachypodium distachyon*, STT3A: XP_003568077.1, STT3B: XP_003580816.1; At, *Arabidopsis thaliana*, STT3A: NP_568380.1, STT3B: NP_174675.2; Sc, *Saccharomyces cerevisiae*, STT3: CAA96722.1

The deduced amino acids of SaSTT3A and SaSTT3B contained the highly conserved STT3 domain and shared about 90 % identity with their homologs from the selected gramineous organisms, such as rice, sorghum and maize. The sequence identity extended over the entire protein instead of certain specific regions (Fig. 1a, b). SaSTT3A and SaSTT3B have 45 % similarity with yeast Stt3p and 80 % similarity with their homologs in *A.thaliana*. Moreover, the characteristic motif of STT3, WWDYG was found in SaSTT3A and SaSTT3B, suggesting the potentially conserved protein function. In yeast, WWDYG likely forms the active site of the enzyme or the binding region of the N-glycosylation recognition site (Yan and Lennarz 2002). The highly conserved WWDYG motif raised up the expectation that SaSTT3A and SaSTT3B could make a similar contribution on substrate recognition and catalysis during N-glycosylation in *S. alterniflora*.

**Fig. 2:**
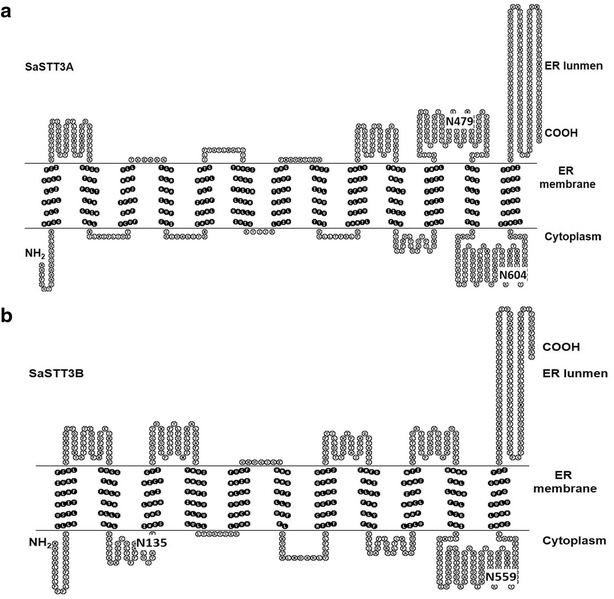
Topology of the proposed SaSTT3A and SaSTT3B in ER membrane. The model was performed using TOPO2 (http://www.sacs.ucsf.edu/TOPO2/) prediction methods. The hydrophobic regions corresponding to transmembrane segments were predicted by the TMpred program (http://www.ch.embnet.org/software/TMPRED_form.html). Putative transmembrane helices (TM) were highlighted in *black*. SaSTT3A had 13 TM helices (**a**) while SaSTT3B had 11 (**b**). NetNGlyc 1.0 Server (http://www.cbs.dtu.dk/services/NetNGlyc/) was used to predict N-glycosylation sites. The locations of putative N-glycosylation sites were shown in *larger characters*. Both SaSTT3A and SaSTT3B carried out a luminal orientation of the C-terminal domain

It has come to be known that the topology of the functional STT3 protein includes a hydrophobic N-terminal domain, 11-13 predicted transmembrane helices and a hydrophilic C-terminal domain located in ER lumen (Kelleher et al. 2003; Kim et al. 2005). The topological analysis of the putative amino acids sequences by TOPO2 server also showed that SaSTT3A and SaSTT3B were membrane proteins and had 13 and 11 transmembrane helices, respectively (Fig. 2a, b). The C terminus of STT3 genes, containing OST active site, was lumenally oriented (Caron et al. 2006). Both SaSTT3A and SaSTT3B were analyzed owning lumenally orienting C terminus, consistent with the previously described (Kim et al. 2005). The similar protein structures of SaSTT3A and SaSTT3B were also shown by Kyte-Doolittle hydropathy profiling (data not shown). Although the topology prediction indicated the SaSTT3 genes were the evolutionally conserved, the precise transmembrane structures of SaSTT3A and SaSTT3B still need a series of in vivo and in vitro topology mapping assays. In addition, we also predicted the potential N-glycosylation sites in both genes. It was found SaSTT3A contained two potential N-glycosylation sites at N479 and N604, and that of SaSTT3B were at N135 and N559 using the NetNGlyc 1.0 Server (Fig. 2a, b).

### Genomic organization of *SaSTT3A* and *SaSTT3B*

**Fig. 3:**
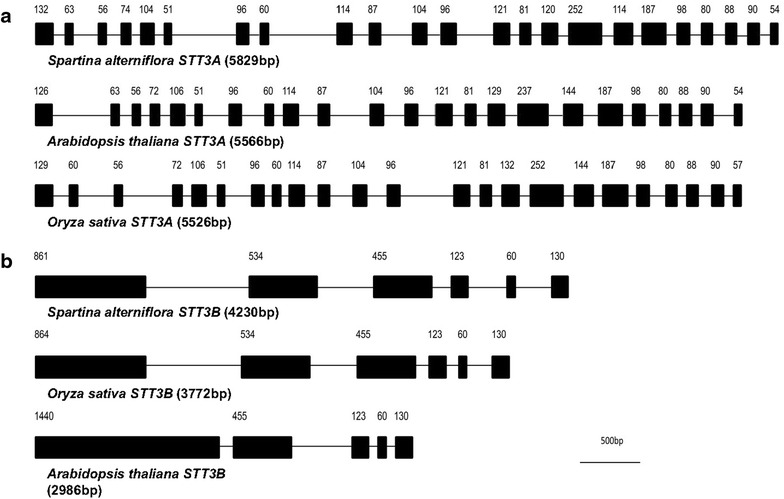
The gene structure and exon organization of predicted *SaSTT3A* and *SaSTT3B.* The genomic organizations of *SaSTT3A* (**a**) and *SaSTT3B* (**b**) with their each counterpart from *A.thaliana* and *O.sativa* were compared. The exons and introns of each gene were represented by *closed boxes* and *lines*, respectively. The *numbers above the boxes* indicated the length of exons in base pairs. Three STT3As were divided into 23 exons but varied in intron size. Three STT3Bs shared similar gene structures except the first exon of *A.thaliana*. The scale of the diagram was given below

The full length of genomic *SaSTT3A* and *SaSTT3B* were characterized using the same strategy as did on CDS and the primers for amplifying genomic fragments were listed in Additional file 1: Table S2. Compared with the coding sequence, the *SaSTT3A* spanned over 23 exons and 22 introns. However, *SaSTT3B* had only 6 exons and 5 introns (Fig. 3). The exon–intron junctions were analyzed and found that all sites followed the consensus rule of the splice acceptor-AG/GT-splice donor for splicing. The precise length of the exons and introns were listed in Table 1. Interestingly, we found *SaSTT3A* and *SaSTT3B* had quite similar gene organization including the number and length of exons to their counterparts in rice and *Arabidopsis* (NP_568380.1, NP_174675.2, NP_001056068.1, NP_001054248.1). The relatively fixed gene structures among the above three organisms further supported that STT3 was evolutionarily conserved.

**Table 1:** Size of exonic/intronic regions and consensus sequences of splicing sites of the *SaSTT3A* and *SaSTT3B*

Gene	Coding exon		5′ Splice donor	3′ Splice donor	Intron	
	No.	Size (bp)			No.	Size (bp)
*SaSTT3A*	1	132	TTCTCC**gt**aaga	gccc**ag**GTAATC	1	100
	2	63	ACTCAG**gt**aagc	tcat**ag**TTTTTG	2	207
	3	56	GACATG**gt**aagc	tttt**ag**GTATCC	3	109
	4	74	TTGGTGgcatgt	tact**ag**GTTGCT	4	72
	5	104	ACAAGA**gt**tgct	ctat**ag**GAAGCA	5	85
	6	51	GCTATG**gt**aaat	taac**ag**GTCCCC	6	513
	7	96	GTAAAG**gt**aatg	atac**ag**ACACTG	7	82
	8	60	TACATG**gt**aaac	tttc**ag**GTCTGC	8	545
	9	114	CCCCTT**gt**aagt	ctgc**ag**GTTATT	9	131
	10	87	TTTCTG**gt**acct	ttgc**ag**GTGTTC	10	258
	11	104	TGGCTT**gt**aagt	ctgc**ag**AGCTGT	11	177
	12	96	TGACCC**gt**aagc	tgac**ag**CACATA	12	305
	13	121	ATCATT**gt**aagt	tcac**ag**TCATGC	13	77
	14	81	GTAATG**gt**aagg	ttca**ag**GTACGG	14	87
	15	129	CCTGCT**gt**acgt	atgt**ag**CCAGGG	15	96
	16	252	TACGTG**gt**aagt	ctgc**ag**GTTCAT	16	97
	17	144	GACAAG**gt**atgc	ctgc**ag**GTTGCT	17	72
	18	187	TTGGAG**gt**tgat	ttac**ag**GGCTTG	18	80
	19	98	TACCTT**gt**gagt	tgcc**ag**AGGGAT	19	101
	20	80	TTACAG**gt**ttgg	ctct**ag**GTTGTG	20	92
	21	88	GAGGAG**gt**aata	ataa**ag**GTTTTC	21	101
	22	90	AAATCA**gt**aagt	ctgc**ag**AATGCC	22	81
	23	54				
*SaSTT3B*	1	861	TTACAG**gt**agtt	ttcc**ag**GTTTTC	1	838
	2	534	GCGAAG**gt**attt	aaac**ag**GGAGCA	2	445
	3	455	TAATAA**gt**aagt	tccc**ag**ATTCTT	3	165
	4	123	CCTAAT**gt**aagt	tggc**ag**GTACAA	4	313
	5	60	TCCAGG**gt**atgt	atgc**ag**ATATGA	5	306
	6	130				

### Phylogenetic analysis

**Fig. 4:**
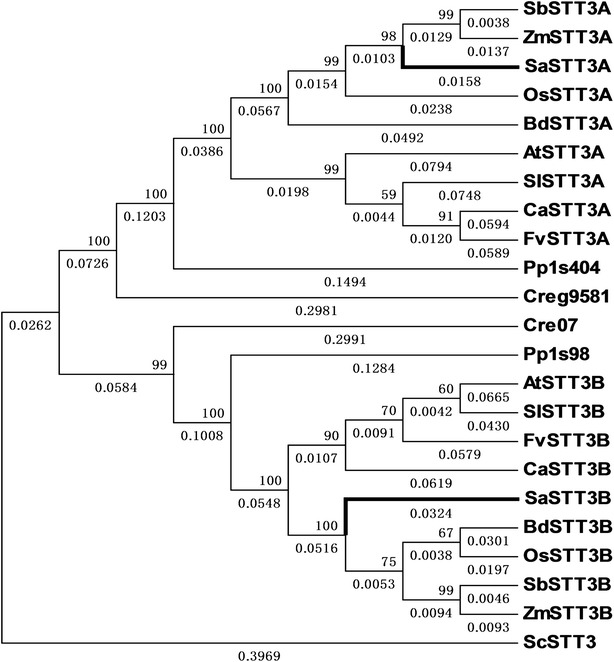
The phylogenetic relationship of STT3A and STT3B. The image was generated by MAGA 5.1 program. Neighbor-Joining STT3 phylogenetic tree showed the relationship between SaSTT3A and SaSTT3B. Branches in *bold* and *labelled* with larger type represented SaSTT3A and SaSTT3B. The values were computed to show the reliability of clustering (above each branch) and genetic distance (below each branch). STT3A and STT3B proteins/homologs from *Fragaria vesca* (FvSTT3A, XP_004287460.1 and FvSTT3B, XP_004299463.1), *Cicer arietinum* (CaSTT3A, XP_004497228.1 and CaSTT3B, XP_004515278.1), *Solanum lycopersicum* (SlSTT3A, XP_004251627.1 and SlSTT3B, XP_004235404.1), *Physcomitrella patens* (Pp1s404: XP_001784419.1 and Pp1s98: XP_001767963.1), *Chlamydomonadales* (Cre07, XP_001690442.1), *Chlamydomonadales* (Creg9581, XP_002955643.1), were analyzed. The accession numbers of SbSTT3A and 3B, ZmSTT3A and 3B, OsSTT3A and 3B, BdSTT3A and 3B, AtSTT3A and 3B and ScSTT3 were the same as stated above

In order to investigate the evolutionary relationship between SaSTT3A and SaSTT3B, a phylogenetic tree was constructed via NJ method. The bootstrap consensus tree was taken inferred from 1000 replicates. The evolutionary distances were marked below the branches representing the number of amino acid substitutions per site. The values above the branches were calculated to evaluate the reliability of the associated taxa clustering. The molecular evolution of STT3s demonstrated the conservation and the correlation with the taxonomy of the selected species. The phylogenetic tree showed SaSTT3A and SaSTT3B evolved in parallel when compared with yeast STT3p and were genetically closer to their each counterparts (Fig. 4). The result indicated SaSTT3A and SaSTT3B could be evolutionarily independent and as a consequence, they might have specialized their own function during the catalysis of N-glycosylation.

### Production of transgenic plants that express SaSTT3A and SaSTT3B

**Fig. 5:**
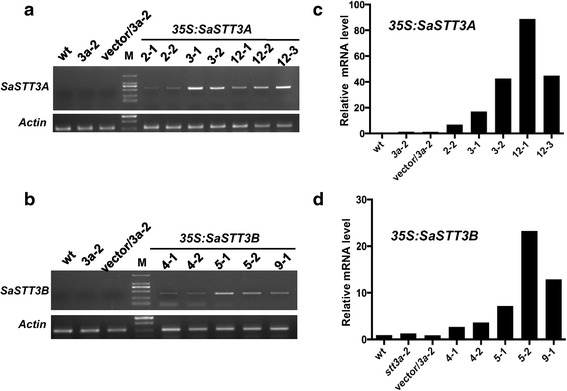
The detection of *SaSTT3A* and *SaSTT3B* expression in *Arabidopsis stt3a*-*2* mutant. The transgenic plants expressing *SaSTT3A* or *SaSTT3B* gene were confirmed using RT-PCR and real-time PCR method. One microgram of total RNA extracted from WT, *stt3a*-*2*, vector control, *35S:SaSTT3A/stt3a*-*2* or *35S:SaSTT3B/stt3a*-*2* seedlings which were grown in the soil for 4 weeks, were used for reverse transcription. **a**, **b** RT-PCR and real-time PCR analysis of *35S:SaSTT3A* transgenic lines. **c**, **d** RT-PCR and real-time PCR analysis of 35S:SaSTT3B transgenic lines

The above analyses revealed that the putative SaSTT3A and SaSTT3B were conserved either in protein sequences or at the gene structure. In order to confirm the function of SaSTT3A and SaSTT3B were truly conserved with their counterparts in the phylogenetic trees, we expressed them in *A. thaliana stt3a*-*2* mutant to observe the phenotypic rescue. It was reported that *A. thaliana stt3a*-*2* mutant was sensitive to high dose of NaCl, typically with growth inhibition of primary roots, swollen root tips, and callose accumulation (Koiwa et al. 2003). The coding sequences of both genes driven either by CaMV 35S or *AtSTT3A* promoter were individually introduced into the *stt3a*-*2* mutant plants via Agrobacterium-mediated transformation. Three T3 transgenic lines of each transform confirmed by RT-PCR (Fig. 5a, c) and real-time PCR (Fig. 5b, d) with *SaSTT3A* or *SaSTT3B* specific primers were selected for further phenotypic observation.

**Fig. 6:**
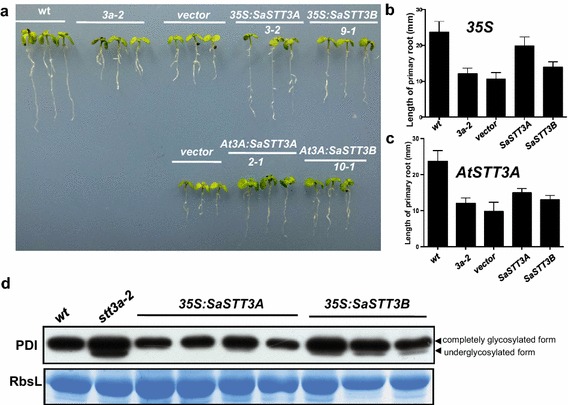
Phenotypic complementation of the *SaSTT3A* on the Arabidopsis mutant. **a** The phenotypic observation of transgenic seedlings grown on 150 mM NaCl stress medium. **b** The statistic analysis of the primary root length of seedlings expressing 3*5S:SaSTT3*. **c** The statistic analysis of the primary root length of seedlings expressing *AtSTT3A:SaSTT3*. **d** Immunoblot analysis of PDI. Total protein extracted from seedlings of WT, *stt3a*-*2*, *35S:SaSTT3A*/*stt3a*-*2*, *35S:SaSTT3B*/*stt3a*-*2* were separated by SDS-PAGE and analyzed by immunoblotting with anti-PDI antibody. The PDI band and underglycosylated protein form were examined and pointed by the *arrows*, respectively. Coommassie *bright blue* stained Rubisco small subunit was used as the loading control

As shown in Fig. 6a, the severely inhibited primary root growth was observed in the *stt3a*-*2* mutants when the high dose of salinity was supplemented in the medium. In contrast to vector control plants, which exhibited obvious stress symptoms grown on the medium supplemented with 150 mM NaCl, *35S:SaSTT3A* plants were conferred salt tolerance phenotypes as WT plants did including the ameliorative root tip phenotype and relieved root growth inhibition. Surprisingly, *SaSTT3B* partially complemented *stt3a* mutation since *35S:SaSTT3B* increased seedlings tolerance against the salt to some extent (Fig. 6a, b). The functional complementation of *SaSTT3* gene driven by the AtSTT3A promoter were also examined. Interestingly, neither *AtSTT3A:SaSTT3A* nor *AtSTT3A:SaSTT3B* worked efficiently on the mutant as they did driven by 35S promoter though they partially recovered the primary root length. The quantitative analysis showed there was no obvious functional difference between *SaSTT3A* and *SaSTT3B* under such condition (Fig. 6c). In order to better understand the functional conservation of SaSTT3A and SaSTT3B, we used the 35S:SaSTT3 transgenic lines for further analysis.

The glycosylation status of PDI (protein disulfide isomerase), which is an endogenous indicator protein sensitive to N-glycosylation occurrence, was examined with western blot method (Hong et al. 2009). Compared to WT, some fractions of PDI under the *stt3a*-*2* background ran faster than in WT, suggesting an underglycosylation status. When *SaSTT3A* was introduced into the mutant driven by 35S, the band was back to the same mobility as that of WT, supporting SaSTT3A has the same function as its homolog in *Arabidopsis*. However, the introduction of SaSTT3B had faint effect on band shift (Fig. 6d). In summary, our result clearly showed SaSTT3A was functionally conserved and it might have the similar function in N-glycosylation. The partial comlementaion of SaSTT3B on *stt3a* phenotype might be ascribed to the specialized function during SaSTT3A and SaSTT3B independent evolution which has been reflected in phylogenetic tree.

## Discussion

In current study, we cloned two members of STT3 family from *S. alterniflora*. Two genes were predicted as ER membrane protein. Sequence alignment revealed that they belonged to STT3A and STT3B cluster respectively and the two genes most likely evolved independently since they located in the different clade on the phylogenetic tree. Both SaSTT3A and SaSTT3B proteins had a typical STT3 domain and a highly conserved WWDYG motif, which raised up the expectation that they would make a contribution to substrate recognition and catalysis of N-glycosylation in *S. alterniflora*. The gene conservation also came from the evidence that SaSTT3A and SaSTT3B had the quite similar gene organization with that of *Arabidopsis* and rice. The further functional analysis showed SaSTT3A could rescue *Arabidopsis stt3a*-*2* salt sensitive phenotype, strongly convincing the STT3 genes evolutionally conserved on N-glycosylation among eukaryotes.

It is noteworthy that there exists at least one member of STT3 family in fully sequenced eukaryotes (Kelleher and Gilmore 2006). STT3 family divides into two branches designated as STT3A and STT3B in more advanced eukaryotes such as plants, insects, and vertebrates according to the evolutionary tree. The results from mammals showed OST complex with STT3A isoform possess a higher selection to the fully assembled N-glycan (Glc_3_Man_9_GlcNAc_2_-PP) than that with STT3B. On the other hand, OST complex containing STT3B shows an increased catalytic rate (Kelleher et al. 2003). In human HeLa cells, Ruiz-Canada et al. uncovered that STT3A and STT3B isoforms were functionally distinct but complementary to insure the maximal efficiency of N-glycosylation, performing co-translational and posttranslational modification, respectively (Ruiz-Canada et al. 2009). It might be possible that STT3A and STT3B act differently on the substrate selection and even the occurrence of N-linked protein modification in plants, which conferred different types of N-glycans on the substrate peptides. Since the biological activity of protein heavily relies on the N-glycan signal (Baisakh 2011; Zandkarimi et al. 2015; Karan and Subudhi 2012), STT3 could be the flexible switch generally affecting N-glycan attachment pattern. Comparably, the mutations involved in N-glycosylation always give rise to invisible defects in plant phenotype under normal growth condition. However, defects in *STT3A* and *CGL1* (*complex glycan less 1*) lead to a hypersensitive phenotype, indicating N-glycosylation modification is crucial for plant tolerance against outer stress. Recently, KOR1/RSW2 (endo-1,4-glucanase KORRIGAN 1/RADIALLY SWOLLEN 2), a membrane glycoprotein responsible for cellulose biosynthesis and necessary for salt tolerance, was reported as a substrate of N-glycosylation and in which N-glycosylation state was regulated by STT3A and CGL1 (Kang et al. 2008; Koiwa et al. 2003). Additionally, mutations in OST3/6 subunit, which facilitates STT3A selecting the N-glycosylation sites of the substrate peptides, lead to a hypersensitive phenotype to salt/osmotic stress (Farid et al. 2013). Thus, N-glycosylation should be of biological significance to protect plants from various stresses, such as salinity. And STT3 as a core element during the N-glycosylation is a link connecting external stress with the functional protein. Since *S. alterniflora* possesses superiority of salt tolerance, it will be necessary to further investigate how the operation and regulation of STT3 coordinate as well as the global N-glycan profile of proteins under salt stress which will provide certain theory reference for the mechanism of plant salt tolerance and elaborate function of N-glycosylation in plant physiology.

## Additional file

**Additional file 1.** In the Supplemental Material Section primers and the corresponding sequences used for gene cloning and plasmid construct are presented.
