# Supplementary material for: Two conserved oligosaccharyltransferase catalytic subunits required for N-glycosylation exist in Spartina alterniflora
Source: Bot Stud. 2015 Nov 11;56:31. doi: 10.1186/s40529-015-0111-9 (PMC5432937; doi:10.1186/s40529-015-0111-9)
Supplement: Supplementary file 1 — Additional file 1. In the Supplemental Material Section primers and the corresponding sequences used for gene cloning and plasmid construct are presented. [file 40529_2015_111_MOESM1_ESM.pptx]

## Slide 1
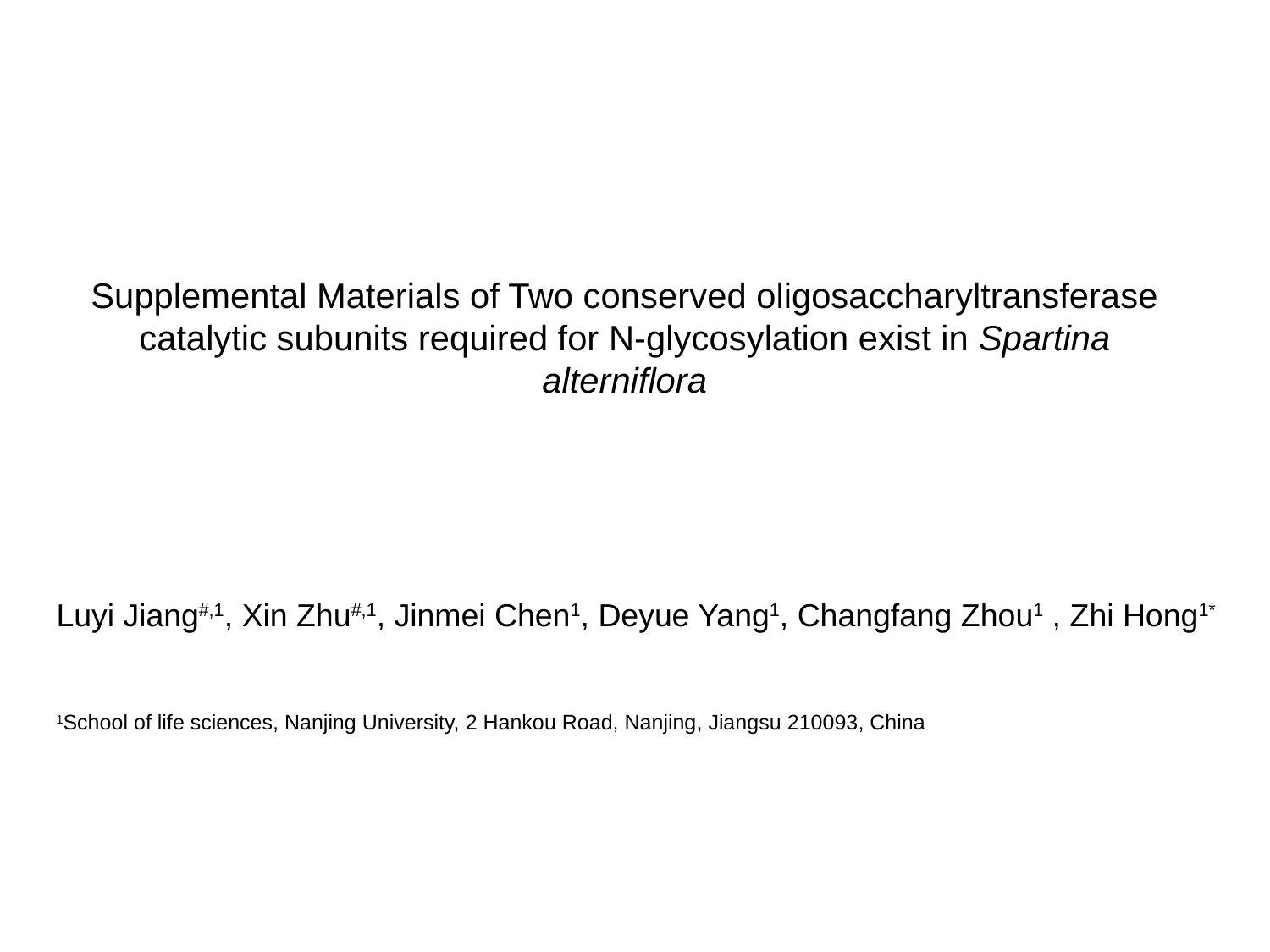

# Supplemental Materials of Two conserved oligosaccharyltransferase catalytic subunits required for N-glycosylation exist in Spartina alterniflora
Luyi Jiang#,1, Xin Zhu#,1, Jinmei Chen1, Deyue Yang1, Changfang Zhou1 , Zhi Hong1*
1School of life sciences, Nanjing University, 2 Hankou Road, Nanjing, Jiangsu 210093, China

## Slide 2
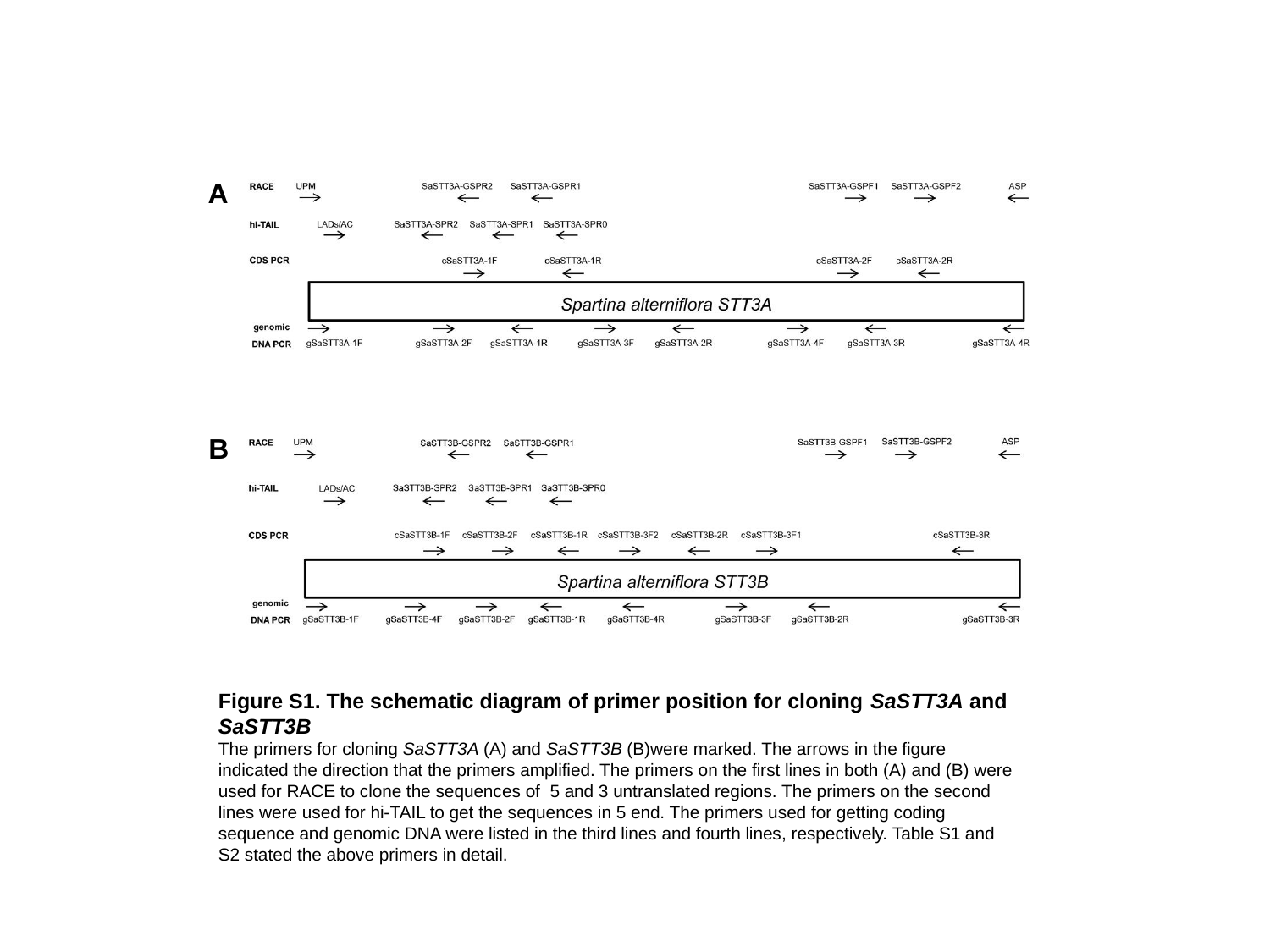

A
B
Figure S1. The schematic diagram of primer position for cloning SaSTT3A and SaSTT3B
The primers for cloning SaSTT3A (A) and SaSTT3B (B)were marked. The arrows in the figure indicated the direction that the primers amplified. The primers on the first lines in both (A) and (B) were used for RACE to clone the sequences of 5 and 3 untranslated regions. The primers on the second lines were used for hi-TAIL to get the sequences in 5 end. The primers used for getting coding sequence and genomic DNA were listed in the third lines and fourth lines, respectively. Table S1 and S2 stated the above primers in detail.

## Slide 3
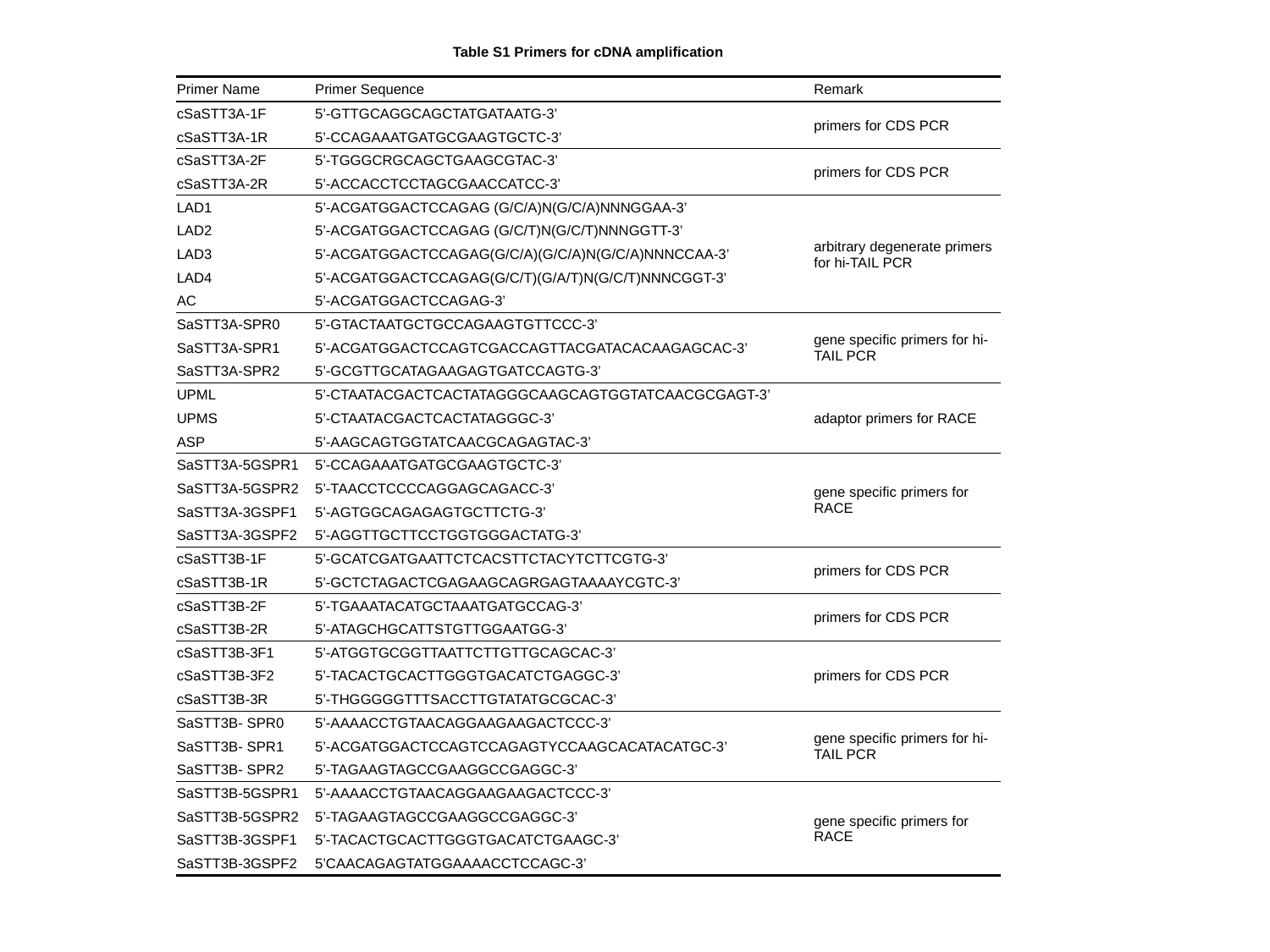

| Table S1 Primers for cDNA amplification | | |
| --- | --- | --- |
| Primer Name | Primer Sequence | Remark |
| cSaSTT3A-1F | 5’-GTTGCAGGCAGCTATGATAATG-3’ | primers for CDS PCR |
| cSaSTT3A-1R | 5’-CCAGAAATGATGCGAAGTGCTC-3’ | |
| cSaSTT3A-2F | 5’-TGGGCRGCAGCTGAAGCGTAC-3’ | primers for CDS PCR |
| cSaSTT3A-2R | 5’-ACCACCTCCTAGCGAACCATCC-3’ | |
| LAD1 | 5’-ACGATGGACTCCAGAG (G/C/A)N(G/C/A)NNNGGAA-3’ | arbitrary degenerate primers for hi-TAIL PCR |
| LAD2 | 5’-ACGATGGACTCCAGAG (G/C/T)N(G/C/T)NNNGGTT-3’ | |
| LAD3 | 5’-ACGATGGACTCCAGAG(G/C/A)(G/C/A)N(G/C/A)NNNCCAA-3’ | |
| LAD4 | 5’-ACGATGGACTCCAGAG(G/C/T)(G/A/T)N(G/C/T)NNNCGGT-3’ | |
| AC | 5’-ACGATGGACTCCAGAG-3’ | |
| SaSTT3A-SPR0 | 5’-GTACTAATGCTGCCAGAAGTGTTCCC-3’ | gene specific primers for hi-TAIL PCR |
| SaSTT3A-SPR1 | 5’-ACGATGGACTCCAGTCGACCAGTTACGATACACAAGAGCAC-3’ | |
| SaSTT3A-SPR2 | 5’-GCGTTGCATAGAAGAGTGATCCAGTG-3’ | |
| UPML | 5’-CTAATACGACTCACTATAGGGCAAGCAGTGGTATCAACGCGAGT-3’ | adaptor primers for RACE |
| UPMS | 5’-CTAATACGACTCACTATAGGGC-3’ | |
| ASP | 5’-AAGCAGTGGTATCAACGCAGAGTAC-3’ | |
| SaSTT3A-5GSPR1 | 5’-CCAGAAATGATGCGAAGTGCTC-3’ | gene specific primers for RACE |
| SaSTT3A-5GSPR2 | 5’-TAACCTCCCCAGGAGCAGACC-3’ | |
| SaSTT3A-3GSPF1 | 5’-AGTGGCAGAGAGTGCTTCTG-3’ | |
| SaSTT3A-3GSPF2 | 5’-AGGTTGCTTCCTGGTGGGACTATG-3’ | |
| cSaSTT3B-1F | 5’-GCATCGATGAATTCTCACSTTCTACYTCTTCGTG-3’ | primers for CDS PCR |
| cSaSTT3B-1R | 5’-GCTCTAGACTCGAGAAGCAGRGAGTAAAAYCGTC-3’ | |
| cSaSTT3B-2F | 5’-TGAAATACATGCTAAATGATGCCAG-3’ | primers for CDS PCR |
| cSaSTT3B-2R | 5’-ATAGCHGCATTSTGTTGGAATGG-3’ | |
| cSaSTT3B-3F1 | 5’-ATGGTGCGGTTAATTCTTGTTGCAGCAC-3’ | primers for CDS PCR |
| cSaSTT3B-3F2 | 5’-TACACTGCACTTGGGTGACATCTGAGGC-3’ | |
| cSaSTT3B-3R | 5’-THGGGGGTTTSACCTTGTATATGCGCAC-3’ | |
| SaSTT3B- SPR0 | 5’-AAAACCTGTAACAGGAAGAAGACTCCC-3’ | gene specific primers for hi-TAIL PCR |
| SaSTT3B- SPR1 | 5’-ACGATGGACTCCAGTCCAGAGTYCCAAGCACATACATGC-3’ | |
| SaSTT3B- SPR2 | 5’-TAGAAGTAGCCGAAGGCCGAGGC-3’ | |
| SaSTT3B-5GSPR1 | 5’-AAAACCTGTAACAGGAAGAAGACTCCC-3’ | gene specific primers for RACE |
| SaSTT3B-5GSPR2 | 5’-TAGAAGTAGCCGAAGGCCGAGGC-3’ | |
| SaSTT3B-3GSPF1 | 5’-TACACTGCACTTGGGTGACATCTGAAGC-3’ | |
| SaSTT3B-3GSPF2 | 5’CAACAGAGTATGGAAAACCTCCAGC-3’ | |

## Slide 4
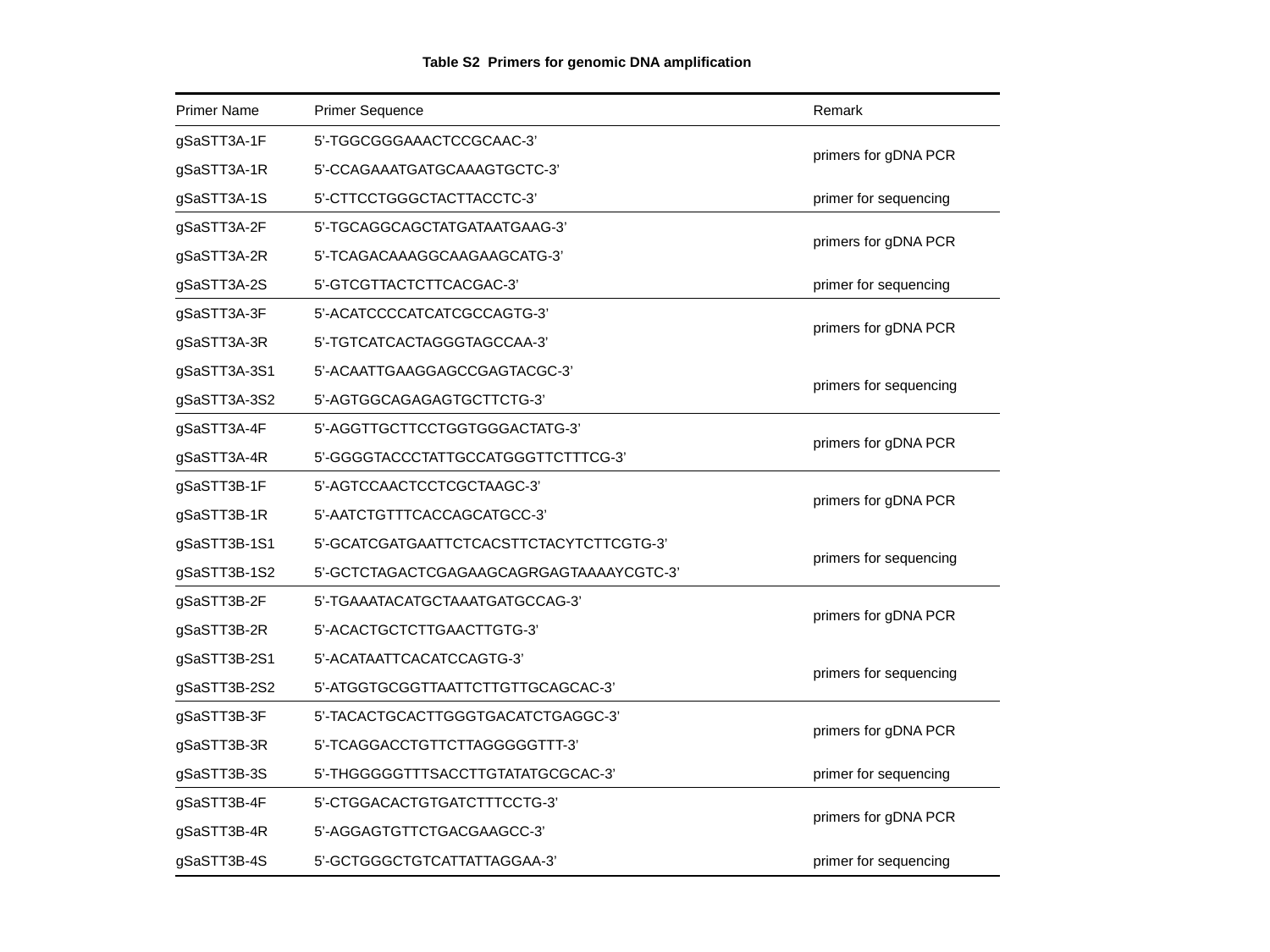

| Table S2 Primers for genomic DNA amplification | | |
| --- | --- | --- |
| Primer Name | Primer Sequence | Remark |
| gSaSTT3A-1F | 5’-TGGCGGGAAACTCCGCAAC-3’ | primers for gDNA PCR |
| gSaSTT3A-1R | 5’-CCAGAAATGATGCAAAGTGCTC-3’ | |
| gSaSTT3A-1S | 5’-CTTCCTGGGCTACTTACCTC-3’ | primer for sequencing |
| gSaSTT3A-2F | 5’-TGCAGGCAGCTATGATAATGAAG-3’ | primers for gDNA PCR |
| gSaSTT3A-2R | 5’-TCAGACAAAGGCAAGAAGCATG-3’ | |
| gSaSTT3A-2S | 5’-GTCGTTACTCTTCACGAC-3’ | primer for sequencing |
| gSaSTT3A-3F | 5’-ACATCCCCATCATCGCCAGTG-3’ | primers for gDNA PCR |
| gSaSTT3A-3R | 5’-TGTCATCACTAGGGTAGCCAA-3’ | |
| gSaSTT3A-3S1 | 5’-ACAATTGAAGGAGCCGAGTACGC-3’ | primers for sequencing |
| gSaSTT3A-3S2 | 5’-AGTGGCAGAGAGTGCTTCTG-3’ | |
| gSaSTT3A-4F | 5’-AGGTTGCTTCCTGGTGGGACTATG-3’ | primers for gDNA PCR |
| gSaSTT3A-4R | 5’-GGGGTACCCTATTGCCATGGGTTCTTTCG-3’ | |
| gSaSTT3B-1F | 5’-AGTCCAACTCCTCGCTAAGC-3’ | primers for gDNA PCR |
| gSaSTT3B-1R | 5’-AATCTGTTTCACCAGCATGCC-3’ | |
| gSaSTT3B-1S1 | 5’-GCATCGATGAATTCTCACSTTCTACYTCTTCGTG-3’ | primers for sequencing |
| gSaSTT3B-1S2 | 5’-GCTCTAGACTCGAGAAGCAGRGAGTAAAAYCGTC-3’ | |
| gSaSTT3B-2F | 5’-TGAAATACATGCTAAATGATGCCAG-3’ | primers for gDNA PCR |
| gSaSTT3B-2R | 5’-ACACTGCTCTTGAACTTGTG-3’ | |
| gSaSTT3B-2S1 | 5’-ACATAATTCACATCCAGTG-3’ | primers for sequencing |
| gSaSTT3B-2S2 | 5’-ATGGTGCGGTTAATTCTTGTTGCAGCAC-3’ | |
| gSaSTT3B-3F | 5’-TACACTGCACTTGGGTGACATCTGAGGC-3’ | primers for gDNA PCR |
| gSaSTT3B-3R | 5’-TCAGGACCTGTTCTTAGGGGGTTT-3’ | |
| gSaSTT3B-3S | 5’-THGGGGGTTTSACCTTGTATATGCGCAC-3’ | primer for sequencing |
| gSaSTT3B-4F | 5’-CTGGACACTGTGATCTTTCCTG-3’ | primers for gDNA PCR |
| gSaSTT3B-4R | 5’-AGGAGTGTTCTGACGAAGCC-3’ | |
| gSaSTT3B-4S | 5’-GCTGGGCTGTCATTATTAGGAA-3’ | primer for sequencing |

## Slide 5
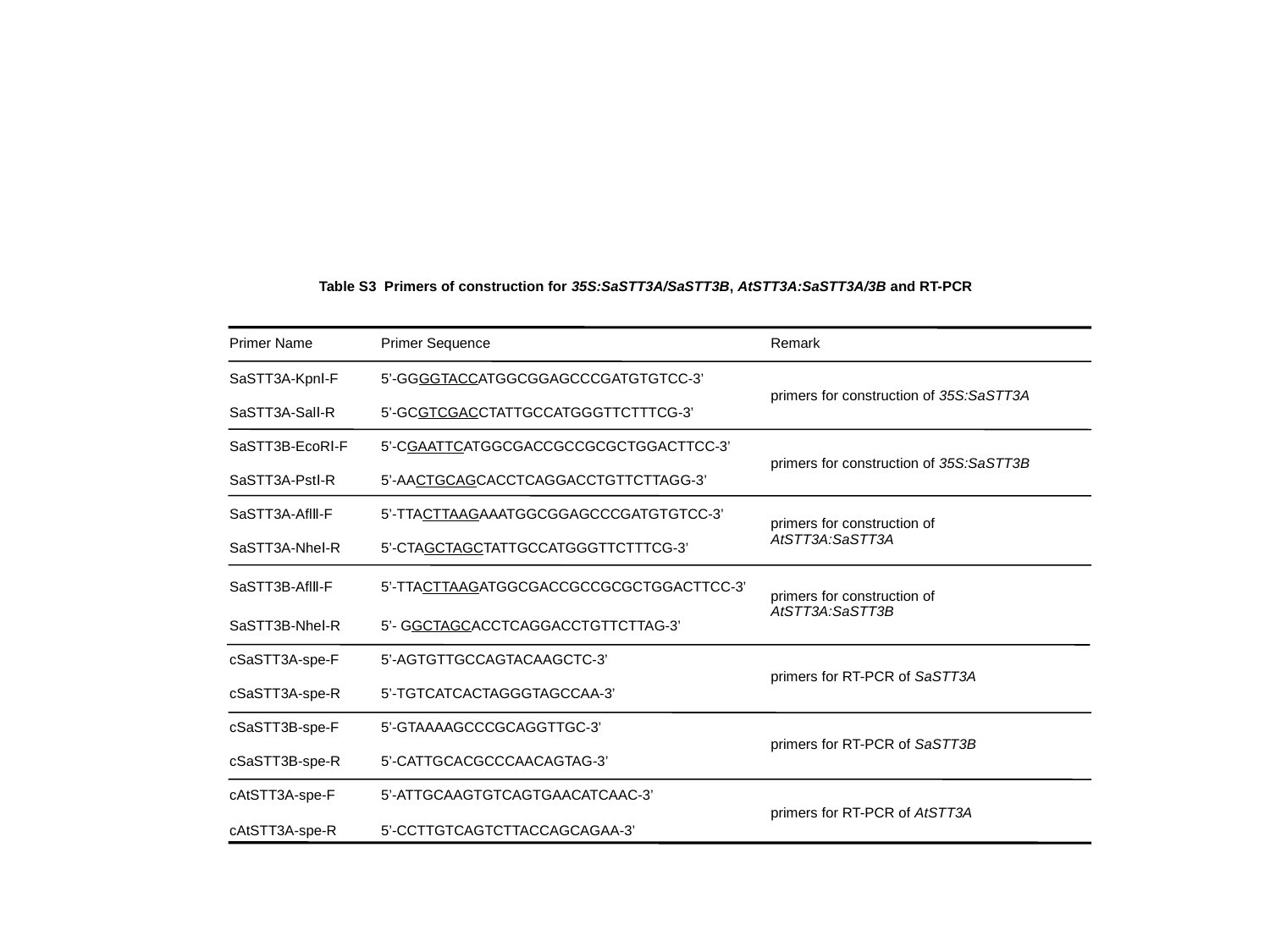

| Table S3 Primers of construction for 35S:SaSTT3A/SaSTT3B, AtSTT3A:SaSTT3A/3B and RT-PCR | | |
| --- | --- | --- |
| Primer Name | Primer Sequence | Remark |
| SaSTT3A-KpnⅠ-F | 5’-GGGGTACCATGGCGGAGCCCGATGTGTCC-3’ | primers for construction of 35S:SaSTT3A |
| SaSTT3A-SalⅠ-R | 5’-GCGTCGACCTATTGCCATGGGTTCTTTCG-3’ | |
| SaSTT3B-EcoRⅠ-F | 5’-CGAATTCATGGCGACCGCCGCGCTGGACTTCC-3’ | primers for construction of 35S:SaSTT3B |
| SaSTT3A-PstⅠ-R | 5’-AACTGCAGCACCTCAGGACCTGTTCTTAGG-3’ | |
| SaSTT3A-AflⅡ-F | 5’-TTACTTAAGAAATGGCGGAGCCCGATGTGTCC-3’ | primers for construction of AtSTT3A:SaSTT3A |
| SaSTT3A-NheⅠ-R | 5’-CTAGCTAGCTATTGCCATGGGTTCTTTCG-3’ | |
| SaSTT3B-AflⅡ-F | 5’-TTACTTAAGATGGCGACCGCCGCGCTGGACTTCC-3’ | primers for construction of AtSTT3A:SaSTT3B |
| SaSTT3B-NheⅠ-R | 5’- GGCTAGCACCTCAGGACCTGTTCTTAG-3’ | |
| cSaSTT3A-spe-F | 5’-AGTGTTGCCAGTACAAGCTC-3’ | primers for RT-PCR of SaSTT3A |
| cSaSTT3A-spe-R | 5’-TGTCATCACTAGGGTAGCCAA-3’ | |
| cSaSTT3B-spe-F | 5’-GTAAAAGCCCGCAGGTTGC-3’ | primers for RT-PCR of SaSTT3B |
| cSaSTT3B-spe-R | 5’-CATTGCACGCCCAACAGTAG-3’ | |
| cAtSTT3A-spe-F | 5’-ATTGCAAGTGTCAGTGAACATCAAC-3’ | primers for RT-PCR of AtSTT3A |
| cAtSTT3A-spe-R | 5’-CCTTGTCAGTCTTACCAGCAGAA-3’ | |
